# Growth Induction and Low-Oxygen Apoptosis Inhibition of Human CD34^+^ Progenitors in Collagen Gels

**DOI:** 10.1155/2013/542810

**Published:** 2012-12-27

**Authors:** Daniele Avitabile, Katrin Salchert, Carsten Werner, Maurizio C. Capogrossi, Maurizio Pesce

**Affiliations:** ^1^Laboratorio di Biologia Vascolare e Medicina Rigenerativa, Centro Cardiologico Monzino, 20138 Milano, Italy; ^2^Laboratorio di Patologia Vascolare, Istituto Dermopatico dell'Immacolata, 00166 Rome, Italy; ^3^Institute of Biofunctional Polymer Material, Leibniz-Institüt für Polymerforschung, 01069 Dresden, Germany; ^4^Laboratorio di Ingegneria Tissutale Cardiovascolare, Centro Cardiologico Monzino IRCCS, Via Parea 4, 20138 Milano, Italy

## Abstract

Various reports have indicated low survival of injected progenitors into unfavorable environments such as the ischemic myocardium or lower limb tissues. This represents a major bottleneck in stem-cell-based cardiovascular regenerative medicine. Strategies to enhance survival of these cells in recipient tissues have been therefore sought to improve stem cell survival and ensure long-term engraftment. In the present contribution, we show that embedding human cord blood-derived CD34^+^ cells into a collagen I-based hydrogel containing cytokines is a suitable strategy to promote stem cell proliferation and protect these cells from anoxia-induced apoptosis.

## 1. Introduction

The success of progenitor cells' transplantation in cardiovascular regenerative medicine depends on efficient engraftment into the hostile ischemic environment [1]. In this framework, cell survival is a relevant issue. In fact, several investigations have shown that injected (stem) cells undergo a relatively rapid clearance from ischemic heart and lower limbs, which may lead to dramatic efficacy reduction. In addition, the absence of suitable conditions to preserve innate repair stem cell function into recipient tissues may lead to adverse effects such as chronic inflammatory responses [2, 3].

The design of advanced biomaterials in cardiovascular regenerative medicine may be a solution to enhance stem cells' engraftment efficiency. In fact, the combination of extracellular matrix components with factors promoting stem cell proliferation and survival may help reproduce a “niche-” like three-dimension (3D) environment allowing optimal engraftment and differentiation, and enhancing their *in vivo* repair function [4–6]. In the present study, we show that addition of a cytokine cocktail to a collagen I-based hydrogel formulation induced proliferation and significantly reduced death of human cord blood-derived CD34^+^ cells cultured in low oxygen, a typical condition encountered into ischemic tissues.

## 2. Materials and Methods

CD34^+^ cells were isolated from human cord blood samples as already established by us [7]; this was performed according to an institutional review board formal approval for cord blood collection (Melzo Hospital; December 12, 2008; authorization no 843). Briefly, cord blood samples were diluted 1 : 3 with phosphate buffered saline (PBS); diluted blood was loaded onto a Ficoll gradient (Lymphoprep) for separating cord blood mononuclear cells. Isolation of CD34^+^ cells was performed using MINI-MACS system (Miltenyi). Collagen I solution was prepared as already described [8]. Briefly, this was performed by neutralizing collagen type I solution (PureCol 3 mg/mL, Nutacon; dissoved in 0.01 N HCl) with 10X phosphate buffered saline (PBS) and 0.1 M NaOH. This liquid working solution (2.4 mg/mL collagen I, PBS 1X, 0.01 M NaOH pH 7.4) was kept on ice until use. Before adding cells, a further dilution of collagen working solution (to a final 1.5 mg/mL concentration) was performed by adding Stem Span culture medium (Stem Cell Technologies) with or without stem cell factor (SCF; 100 ng/mL), interleukin-6 (IL6; 20 ng/mL), interleukin-3 (IL3; 20 ng/mL), and fetal liver tyrosine kinase 3-ligand (Flt3-L; 100 ng/m), all from Stem Cell Technologies. CD34^+^ cells (2 × 10^7^ cells/mL in 10 *μ*L PBS) were finally added to the collagen-cytokines mixture and gently mixed to allow a homogeneous distribution. Matrix/cell suspensions were then incubated at 37°C in a humidified chamber for 15 minutes, after which they were transferred into 24 well plates (Costar); the formation of a three-dimensional semisolid collagen matrix was completed in these wells after further 45 minutes at 37°C. Each well containing CD34^+^ cells seeded matrix (3D condition) was, finally, covered with Stem Span alone or containing the cytokines mix at the same concentration used in the matrix. As a control, the same number of cells was cultured in normal liquid culture using the same medium and cytokines concentration (2D condition). To assess cell phenotype after culturing in 3D conditions, collagen gel was digested using collagenase IV solution, before recovering cells by centrifugation, staining with monoclonal antibodies against CD14, CD31, CD34, and CD133 antigens (all from BD Biosciences), and analysis with a FACScalibur (BD Biosciences) flow cytometer. To perform culture in hypoxia, culture plates were placed into a humidified hypoxia chamber, hermetically sealed, and perfused with a 95% N_2_/5% CO_2_ atmosphere. After 7 days, cells were recovered by collagenase IV digestion and immediately stained for annexin V (AnnV) and propidium Iodide (PI) to detect apoptosis.

## 3. Results and Discussion

**Figure 1:**
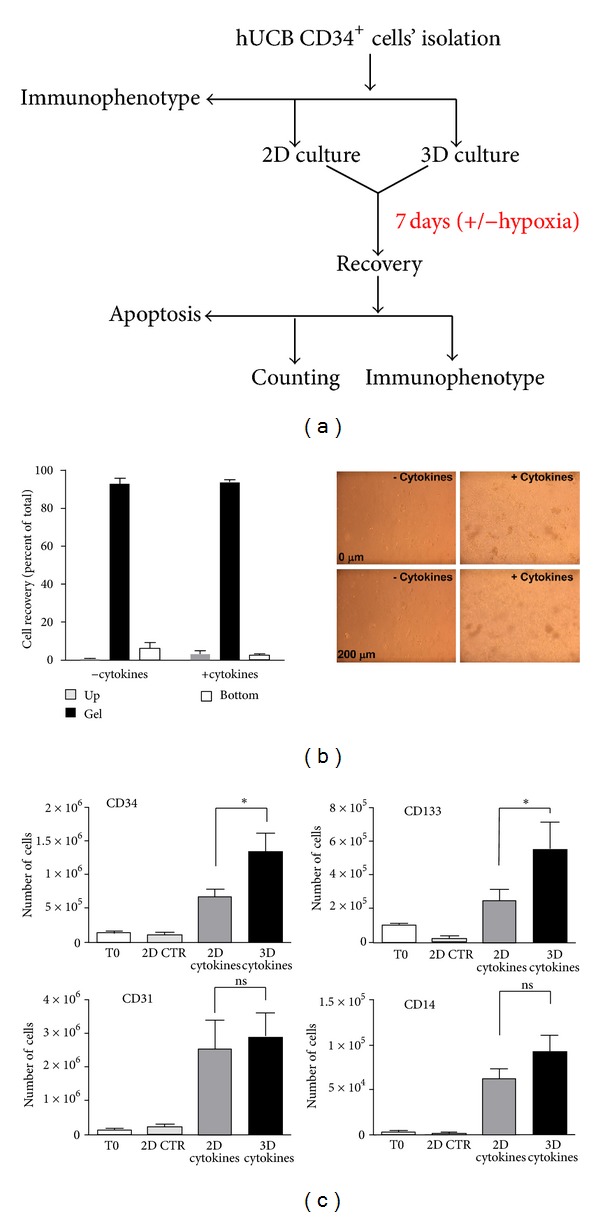
Experimental scheme and results of 3D matrix culture. (a) Procedures used in the present study with all the principal actions performed to assess cell phenotype, growth, and survival. (b) Counting of recovered cells from the bottom of culture plates (bottom), 3D matrix (gel), and the overlying cell culture medium (up) revealed that the majority of cells remained confined in the matrix; data are expressed as percentage of the total number of cells recovered from the three compartments in the presence or the absence of cytokines. Micrographs on the right show the presence of isolated cells (− cytokines) or proliferating cells clusters (+ cytokines) at different focal planes (*Z*-axis distance 200 *μ*m) in the 3D matrix. (c) Flow cytometry experiments showed that, at seven days of culture, the number of cells expressing CD34 and CD133 stem cell markers was significantly enhanced by culture in 3D conditions compared to 2D in the presence of the same cytokines' concentration. By contrast, the number of CD31 and CD14 cells was not significantly changed. * indicates *P* < 0.05 by one-way ANOVA with Newman-Keuls post-hoc analysis (*n* = 4).

Figure 1(a) shows the experimental scheme adopted to perform our tests. After an initial preexpansion for two days in liquid culture, cells were cultured for one week in 2D and 3D conditions.  Figure 1(b) shows that the majority of cells recovered from culture wells by hydrogel digestion were entrapped in the collagen matrix. Moreover, presence of cytokines induced the formation of typical clusters of rapidly expanding cells, present at different focus planes in the matrix. To screen for differences in the growth of cells in the presence or the absence of cytokines in 2D and 3D conditions, a flow cytometry analysis of cells recovered after 7 days of culture was performed.  Figure 1(c) shows the number of cells expressing typical “early” endothelial progenitor cells (EPCs) markers CD34, CD133, CD31, and CD14 [1, 9], also in comparison with that of cells cultured in 2D conditions for 7 days in the absence of cytokines (2D CTR). The presence of cytokines in the matrix promoted a net increase in the number of immature cells, as revealed by the significant elevation of CD34^+^ and CD133^+^ cells in 3D versus 2D. Interestingly, the number of cells expressing CD31, a typical endothelial cells marker, was similarly increased in 2D and 3D, while the number of CD14^+^ monocytes was only marginally changed. Taken together, these results suggest that presence of cytokines in the 3D environment favors the expansion of immature EPCs, without increasing their monocyte commitment.

**Figure 2:**
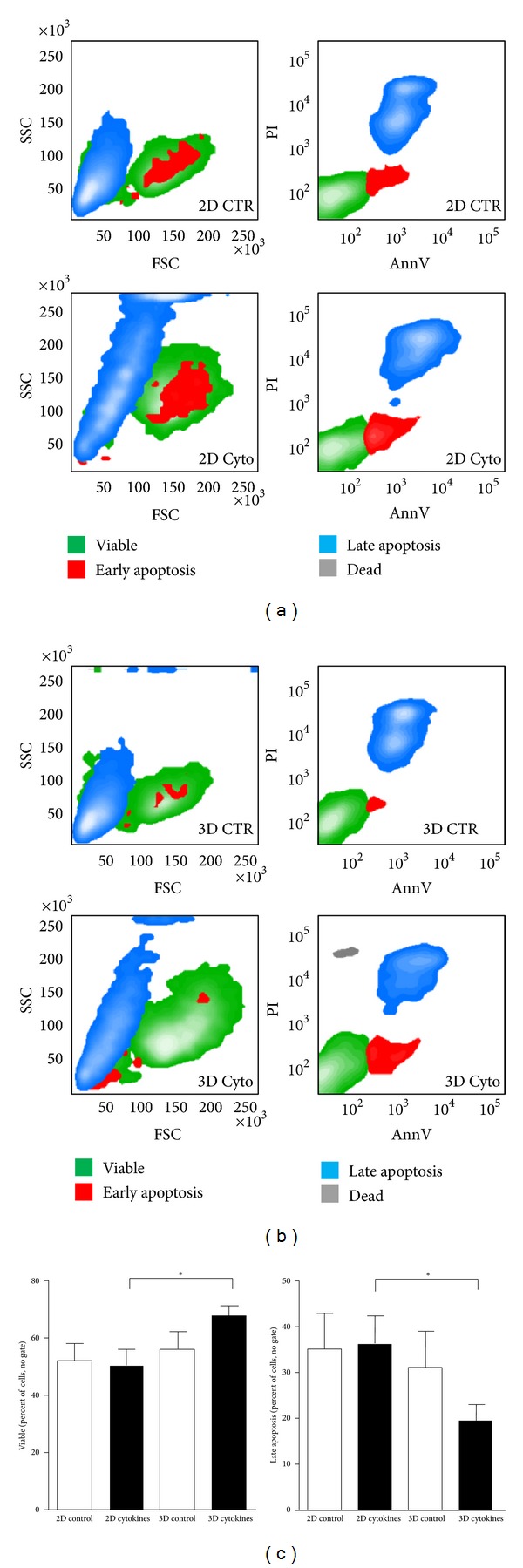
Increased survival of 3D versus 2D cultured CD34+ cells under severe hypoxia conditions. (a-b) Representative flow cytometry contour plots showing the physical appearance (FSC-SSC plot) and the annexinV/PI staining of CD34+ cells cultured for 7 days in an atmosphere containing 95%N_2_/5%CO_2_. Multicolor staining of the different regions indicates, respectively, viable cells (green), early apoptotic cells (red), late apoptotic cells (blue), and necrotic cells (gray). (c) Quantification of viable and late apoptosis cells. No significant differences were observed in viable and late apoptotic cells' number in 2D cultures (±cytokines) and 3D culture without cytokines; by contrast, the number of viable cells was significantly increased and that of late apoptotic cells was significantly reduced, by cytokines-supplemented matrix culture. * indicates *P* < 0.05 by paired Student's *t*-test (3D cytokines versus 3D control); *n* = 5.

Exposure to lower than 20% oxygen is a strategy to enhance formation of endothelial cells' colonies (CFU-EC) by immature bone-marrow-derived CD133^+^ cells [10] or to preserve clonogenic ability of human hematopoietic progenitors [11]. In fact, oxygen level around or below 1% is a concentration normally experienced by the most immature hematopoietic progenitors in the bone marrow niche, where it appears a critical component of stemness maintenance [12, 13]. On the other hand, exposure of immature progenitors to anoxia, such as in the ischemic myocardium, has been also reported to enhance death due to induction of apoptosis [14]. To explore whether CD34^+^ cells undergo cell death when cultured in oxygen deprivation conditions, cultures in anoxia chambers were set up, followed by AnnV/PI staining and flow cytometry [15]. As shown in Figure 2, cells cultured in 2D for 7 days in anoxia, either in the presence or the absence of cytokines, maintained a viability around 60%. In addition, they showed a similar percentage of cells in early apoptosis (AnnV^+^/PI^−^) and late apoptosis (AnnV^+^/PI^+^) stages. By contrast, culture in 3D conditions for the same time extent improved survival to about 80% and reduced the percentage of cells in late apoptosis. Interestingly, the effect of 3D culture was observed only in the cytokines-containing condition, while embedding in the hydrogel alone was not sufficient to rescue cells from low-oxygen-induced cell death, indicating the 3D/cytokines combination as the most powerful treatment preserving CD34^+^ cells viability.

## 4. Conclusions

The results shown in the present study show that embedding of primary CD34^+^ progenitors into cytokines-containing 3D matrices is a suitable strategy to maximize expansion under normal oxygen content and to protect them from low-oxygen-induced apoptosis. These effects may result from an optimal cytokine “presentation” in the 3D environment compared to liquid cultures [16] and/or from a synergistic action of cytokine-dependent prosurvival pathways, cell proliferation signaling, and matrix-dependent cues [17]. Whether or not culturing in 3D collagen gel has an influence on CD34^+^ cell functions after culturing in low oxygen, is currently being investigated in our laboratory. However, the relative ease of the collagen I mixture preparation and its formulation with components which are readily available for human use suggests that the embedding method shown here may be used to maximize stem cell transplantation efficiency in patients with heart and peripheral ischemia or to optimize stem cells seeding into biomaterials for the production of next generation bioartificial tissues.
